# Examining Different Analysis Protocols Targeting Hospital Sanitary Facility Microbiomes

**DOI:** 10.3390/microorganisms11010185

**Published:** 2023-01-11

**Authors:** Claudio Neidhöfer, Esther Sib, Al-Harith Benhsain, Christina Mutschnik-Raab, Anna Schwabe, Alexander Wollkopf, Nina Wetzig, Martin A. Sieber, Ralf Thiele, Manuel Döhla, Steffen Engelhart, Nico T. Mutters, Marijo Parčina

**Affiliations:** 1 Institute of Medical Microbiology, Immunology and Parasitology, University Hospital Bonn, Venusberg-Campus 1, 53127 Bonn, Germany; 2 Institute for Hygiene and Public Health, University of Bonn, 53127 Bonn, Germany; 3 Hospital Pharmacy, Bundeswehr Central Hospital Koblenz, 56072 Koblenz, Germany; 4 Institute for Functional Gene Analytics, Bonn-Rhein-Sieg University of Applied Sciences, 53757 Sankt Augustin, Germany; 5 Department of Microbiology and Hospital Hygiene, Bundeswehr Central Hospital Koblenz, 56072 Koblenz, Germany

**Keywords:** NGS, high-throughput DNA sequencing, 16S rRNA gene sequencing, microbiome, microbial ecology, built environment, hospital environment, hospital-acquired infections

## Abstract

Indoor spaces exhibit microbial compositions that are distinctly dissimilar from one another and from outdoor spaces. Unique in this regard, and a topic that has only recently come into focus, is the microbiome of hospitals. While the benefits of knowing exactly which microorganisms propagate how and where in hospitals are undoubtedly beneficial for preventing hospital-acquired infections, there are, to date, no standardized procedures on how to best study the hospital microbiome. Our study aimed to investigate the microbiome of hospital sanitary facilities, outlining the extent to which hospital microbiome analyses differ according to sample-preparation protocol. For this purpose, fifty samples were collected from two separate hospitals—from three wards and one hospital laboratory—using two different storage media from which DNA was extracted using two different extraction kits and sequenced with two different primer pairs (V1–V2 and V3–V4). There were no observable differences between the sample-preservation media, small differences in detected taxa between the DNA extraction kits (mainly concerning *Propionibacteriaceae*), and large differences in detected taxa between the two primer pairs V1–V2 and V3–V4. This analysis also showed that microbial occurrences and compositions can vary greatly from toilets to sinks to showers and across wards and hospitals. In surgical wards, patient toilets appeared to be characterized by lower species richness and diversity than staff toilets. Which sampling sites are the best for which assessments should be analyzed in more depth. The fact that the sample processing methods we investigated (apart from the choice of primers) seem to have changed the results only slightly suggests that comparing hospital microbiome studies is a realistic option. The observed differences in species richness and diversity between patient and staff toilets should be further investigated, as these, if confirmed, could be a result of excreted antimicrobials.

## 1. Introduction

The fight against antibiotic-resistant pathogens has led to an ever-increasing burden on the healthcare system in recent decades. A lack of effective treatment inevitably leads to a vicious cycle of prolonged hospitalization, which in turn fosters hospital-acquired infections with pathogens that may also be highly resistant to therapy, and ultimately, to a concomitant surge in morbidity and mortality [1]. In 2021, the WHO emphasized the importance of global surveillance of antimicrobial resistance to effectively implement strategies to combat MDR pathogens [2]. Next-generation sequencing has been widely used for this purpose over the past decade and shows great promise [3]. The obtained data are generally used to determine antimicrobial resistance, virulence patterns, and clonality [3,4,5]. In contrast, the idea of studying the totality of microorganisms in patients and their hospital environments is a more recent one and opens up the possibility of better understanding the emergence of niches of multidrug-resistant organisms in the patient and the hospital [6,7,8].

At any time, we are surrounded by microbes that can positively and negatively influence our health. The totality of microorganisms we carry in and on us is often categorized as our microbiome and thus distinguished by definition from microorganisms that merely surround us. To what extent some should be called our own and others not remains part of many ongoing investigations [8,9,10,11,12]. Throughout modern human evolution, built environments—and hospitals in particular—have changed in ways that make them increasingly inhospitable to microbial life, with largely dry surfaces often covered with antimicrobial materials [13]. While this has undoubtedly contributed to reducing the spread of communicable diseases, it has also changed our microbial relationship with the environment [13,14]. Research concerning the microbiome of indoor environments such as hospitals, houses, or buildings could have several implications for human health [7,8,9,10,11,12,13,14]. The hospital microbiome consists of complex, nested systems with a multilayered transmission of strains, plasmids, and smaller genetic elements between patients, medical staff, hospital surfaces, and water networks [15]. Although metagenomic analyses can be used beyond bacterial community relations to obtain information on non-bacterial microorganisms and resistance genes, amplicon sequencing of genes such as the 16S rRNA gene offers the best cost–benefit ratio for assessing indoor microbiome profiles to date [16]. The currently available data suggest that NGS bacteriome analysis (together with a similar assessment of the mycobiome and the resistome) provides valuable additional information about the microbiome contaminating the hospital environment, resulting in a subsequent improvement in protocols and measures to combat the increasing prevalence of antimicrobial resistance [13,15,17]. These molecular analyses could ideally be integrated into ongoing surveillance programs. Further research and technological advances are needed before these approaches can be routinely used for hospital surveillance; however, their ability to track outbreaks of multidrug-resistant bacteria and the spread of antimicrobial resistance, identify persistent environmental reservoirs, and assess future risks [7,8,12,13,14,15,16,17] is promising.

In our study, we compared different sample collection sites in hospital-patient bathrooms and different sample preparation and sequencing protocols regarding the number and type of detected taxa. This was performed to investigate the microbiomes of hospital sanitary facilities and evaluate the degree to which hospital microbiome analyses vary with different protocols.

## 2. Materials and Methods

### 2.1. Sample Collection

Two hospitals were included in this study. One is a tertiary referral and maximum-care facility (TCH), and the other a military hospital (MH). The two analyzed hospitals are approximately 60 km apart. Altogether, 50 sites were sampled. Each site was swabbed and preserved in 1 mL eNAT medium tubes (Copan, Brescia, Italy) and 1 mL DNA/RNA Shield Collection Tubes (Zymo Research Europe GmbH, Freiburg, Germany), totaling 100 samples. The 50 sample sites included biofilms along the water level of 24 toilets (12MH/12TCH) and in siphons of 14 wash basins (12MH/2TCH) and 12 showers (all MH).

### 2.2. DNA Extraction

Samples were stored for less than 14 days at 4 °C before highly purified DNA was extracted using both the column-based PureLink Microbiome DNA Purification Kit (Thermo Fisher Scientific, Waltham, MA, USA), henceforward referred to as the PMP kit, and the ZymoBIOMICS DNA Miniprep Kit (Zymo Research Europe GmbH, Freiburg, Germany), henceforward referred to as the ZBM kit, according to the manufacturers’ instructions. At the end of the extraction process, the DNA was qualitatively and quantitatively evaluated using the NanoDrop OneC (Thermo Fisher Scientific, Waltham, MA, USA).

### 2.3. Library Preparation

The 16S rRNA gene sequencing libraries were constructed using the Quick-16S NGS Library Prep Kit (Zymo Research Europe GmbH, Freiburg, Germany) with its included optimized primer pairs. All samples extracted with the PMP kit and 91 of those extracted with the ZBM kit were sequenced with the V1–V2 primer pairs, whereas 89 samples extracted with the PMP kit and 20 of those extracted with the ZBM kit were sequenced with the V3–V4 primer pairs. Each run included 94 samples, the positive control included in the kit, and a negative control. For quantitative PCR, quality control, and normalization purposes, the Bio-Rad CFX96 Real-Time PCR Detection System (Bio-Rad Laboratories, Inc., Hercules, CA, USA) was utilized. 

### 2.4. Sequencing

After pooling, the DNA was quantified with the QuantiFluor dsDNA System on the Quantus Fluorometer (Promega GmbH, Walldorf, Germany) and diluted strictly according to the Illumina protocol for MiSeq sample preparation. For the final library, a loading concentration of 10 pM was chosen and a 10% Illumina v3 PhiX spike-in control added before running it on the Illumina MiSeq platform. Libraries prepared using the V1–V2 primer pair were sequenced with 500cycle v2 Illumina MiSeq Reagent Kits, and libraries prepared using the V3–V4 primer pair with 600cycle v3 Illumina MiSeq Reagent Kits. All reagents and equipment for sequencing samples were obtained from Illumina, San Diego, CA, USA.

### 2.5. Bioinformatic Analysis

The bioinformatics analysis included three main parts, starting with the preprocessing of raw paired-end reads. Following preprocessing, the sequences were assigned to taxonomies. Finally, a statistical and graphical evaluation was performed on the resulting taxa. QIIME2 (2022.8) [18] was used for both preprocessing and classification of the data. With the plugin tool DADA2 (2022.8.0) [19], forward and reverse reads were trimmed from the 3′ end at position 249, while shorter reads and low-quality reads were discarded. DADA2 was also used to perform error correction, the merging of forward and reverse reads if there was an overlap of at least 12 base pairs, and chimera removal. The processed sequences were clustered into operational taxonomic units (OTUs) of 100% sequence identity and assigned to taxa using a classifier trained on full-length sequences of SILVA [20]. The trained classifier was provided by QIIME2 using scikit-learn 0.24.1 and the plugin tool q2-feature-classifier [21,22]. Based on the quantified OTUs and taxa, different diversity indices were calculated using Python and the skbio.diversity library: the richness, Shannon, Simpson, and Fisher indices as a measurement for alpha diversity, and the Bray–Curtis and Jaccard indices as a measurement for beta diversity. 

All data relevant to this study are included in this article.

## 3. Results

**Figure 1:**
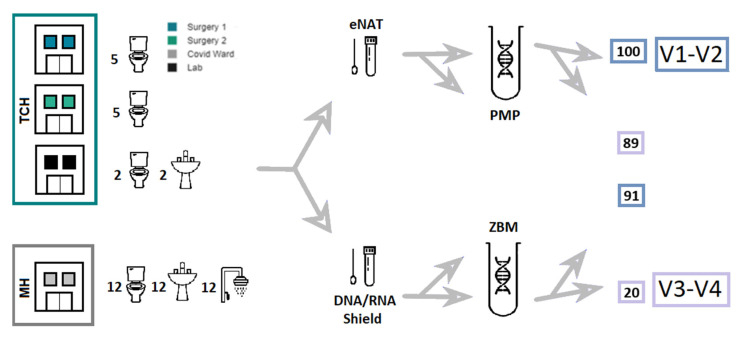
Specimen collection, preservation, and processing flow. The color of the boxes on the right of the image indicates how many samples from each workflow were ultimately sequenced with the corresponding primers.

In total, 300 sequenced hospital microbiome profiles (which generated a total of 29,774,051 reads with a mean read count of 99,247 per microbiome) passed our set minimum quality criteria of >4000 reads and >1400 merged reads each. At the phylum-to-species level, all taxa with an average prevalence of >0.3% were considered for the statistical analysis. These included 13 phyla, 15 classes, 34 orders, 47 families, 50 genera, and 3 species. Swabs from 50 sites, each in 2 different storage media, yielded 100 samples. DNA was extracted from 92 samples using both the ZBM and PMP kits and from the remaining 8 with only the PMP kit, which resulted in 192 extracted DNA eluates. Of these, 108 were sequenced with the V1–V2 and V3–V4 primer pairs. Of the remaining 84, 83 were sequenced with only the V1–V2 primer pairs and 1 with only the V3–V4 primer pairs (resulting in 300 microbiome profiles). (See Figure 1.) This analysis showed, and the manufacturer subsequently confirmed, that 10 of the Shield tubes were affected by the contamination of a raw chemical during production, which ultimately limited the final evaluation to 262 microbiome profiles. The remaining Shield tubes belonged to another batch.

### 3.1. Present Taxa

**Figure 2:**
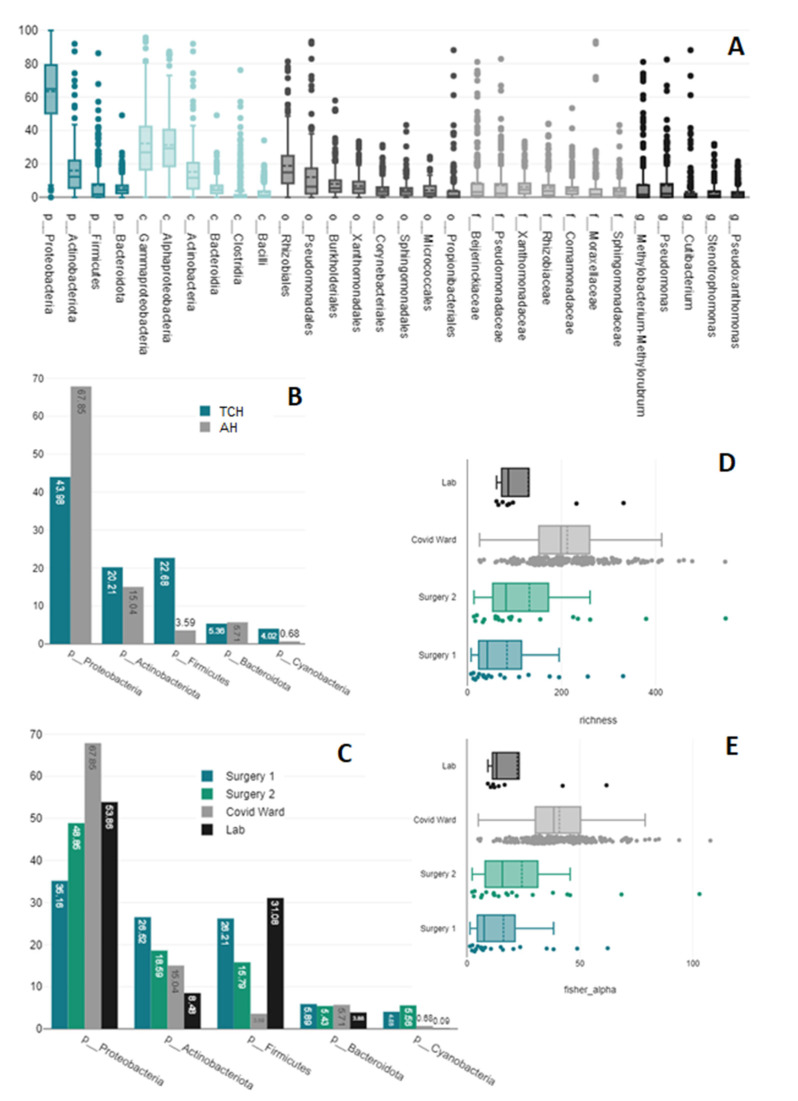
(**A**) Overall prevalence of the 30 most important taxa. Colors allow a more straightforward distinction between the different levels of taxonomic resolution. (**B**) Prevalence of the five most important phyla in the two hospitals and (**C**) institutes/wards. (**D**) Differences in richness and (**E**) Fisher-alpha diversity across the different institutes/wards.

Altogether, 99.99% of the reads were of bacterial origin, and 0.01% were archaeal. The latter were only found in one toilet (with both primer pairs) and one shower (only V1–V2). The prevalence of the 30 most important taxa is displayed in Figure 2A. Only three taxa with an average prevalence of >0.3% were identified at the species level—*Acinetobacter ursingii*, *Lactobacillus iners,* and *Microbacterium lacticum*—the first of which was only detected in one hospital (MH) (t(212) = −3.33, *p* = 0.001). In terms of detected phyla, richness, and diversity, the most striking differences were observed between the two hospitals and the institutes/wards (see Figure 2B–E).

### 3.2. Collection and Preservation Systems

Given that among swabs obtained in surgical wards, only those collected with eNAT were valid and taxa and diversity differed distinctively between institutes, these wards were not considered for comparing the two collection and storage systems. This left 221 microbiomes for comparison (111 Shield, 110 eNAT). No statistically significant differences in terms of detected phyla, classes, orders, families, or species were detected between the two different systems. No statistically significant differences were detected with respect to richness or any of the three diversity indices (Shannon, Fisher-alpha, Simpson).

### 3.3. DNA Extraction

Because many of the samples extracted with the PMP kit were also sequenced with the V3–V4 primer pair in addition to the V1–V2 primer, only samples sequenced with the latter were selected for a first comparison of the two kits, resulting in an analysis that included 164 microbiomes (82 PMP, 82 ZBM). Among the samples extracted with the PMP kit, a significantly higher prevalence of the phylum *Actinobacteriota* (t(162) = −2.1, *p* = 0.037), the class *Actinobacteria* (t(162) = −2.04, *p* = 0.043), the order *Propionibacteriales* (t(162) = −2.01, *p* = 0.046), and the family *Propionibacteriaceae* (t(162) = −1.99, *p* = 0.048) was observed. No statistically significant differences were detected concerning richness or diversity. 

A second analysis of the two kits included samples sequenced with the V3–V4 primer pair and extracted with both extraction kits, resulting in an analysis that included 20 microbiomes (10 PMP, 10 ZBM). Among the samples extracted with the PMP kit, a significantly higher prevalence of the phyla *Actinobacteriota* (t(18) = −2.71, *p* = 0.014) and *Verrucomicrobiota* (t(9) = −2.38, *p* = 0.041), the class *Actinobacteria* (t(18) = −2.23, *p* = 0.039), the order *Propionibacteriales* (t(18) = −2.11, *p* = 0.049), the family *Propionibacteriaceae* (t(18) = −2.43, *p* = 0.026), and the genus *Mycobacterium* (t(9) = −2.55, *p* = 0.031) was observed. On the contrary, a lower prevalence was observed of the order *Pseudomonadales* (t(12.29) = 2.34, *p* = 0.037), the family *Pseudomonadaceae* (t(9.45) = 2.35, *p* = 0.042), and the genus *Pseudomonas* (t(9.45) = 2.35, *p* = 0.042).

### 3.4. Primer Pairs

Of all 262 microbiomes, 172 were sequenced with V1–V2 primer pairs and 89 with V3–V4 primer pairs. On average, microbiomes sequenced with V1–V2 primers were found to be richer (t(259) = 2.09, *p* = 0.038) and more diverse in terms of their Shannon Diversity Index (t(259) = 2.4, *p* = 0.017). To analyze differences in the detection and prevalence of specific taxa, we restricted the comparison to microbiomes sequenced with V1–V2 and V3–V4 primers. This left us with 181 microbiomes (92 V1–V2, 89 V3–V4), among which the differences in terms of richness and diversity were not confirmed. Which taxa were detected more or less with which primer pair is shown in Table 1.

### 3.5. Sampling Sites

**Figure 3:**
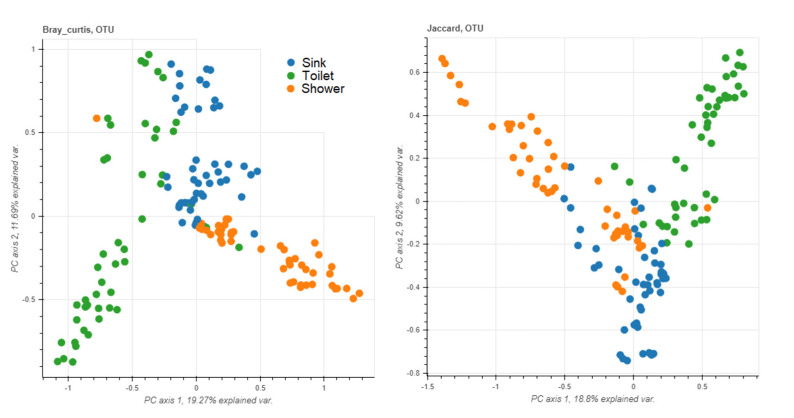
Principal coordinate analysis (PCoA) plot with Bray–Curtis (**left**) and Jaccard (**right**) dissimilarity highlighting operational taxonomic unit (OUT) differences and similarities between MH samples linked to the sampling site.

In order to assign the prevalence of different taxa to specific swab sites (toilets/sinks/showers), we compared the microbiomes of different sites in the most suitable ward (COVID ward). Our analysis was limited to one ward due to considerable discrepancies between the other wards, previously shown in Figure 1, and because it was the only ward in which toilets, sinks, and showers were sampled. We also limited our analysis in each case to only samples stored in one of the two media (eNAT), extracted by one of the two extraction methods (PMP), and sequenced with one of the two primer pairs (V1–V2) and then confirmed the results with those of the other combinations (all groups n = 34 to 36). The reported *p*-values refer to the significance of the analysis of variance in the group of samples stored in eNAT, extracted with the PMP kit, and sequenced with the V1–V2 primer pair. Where ANOVA found significant differences, a Bonferroni post hoc test was used to compare the groups in pairs. The results, confirmed across all groups of samples, included a significantly higher prevalence of the family *Pseudomonadaceae* (F = 7.79, *p* = 0.002) and the genus *Pseudomonas* (F = 7.64, *p* = 0.002) in showers; a higher prevalence of the genera *Acinetobacter* (F = 8.45, *p* = 0.001) and *Phenylobacterium* (F = 4.18, *p* = 0.024) in sinks; and a higher prevalence of the families *Hyphomicrobiaceae* (F = 11.58, *p* = < 0.001) and *Beijerinckiaceae* (F = 3.87, *p* = 0.031) and the genera *Hyphomicrobium* (F = 13.19, *p* = < 0.001) and *Methylobacterium*/*Methylorubrum* (F = 3.89, *p* = 0.03) in toilets. No significant differences were detected regarding taxa richness or diversity. Figure 3 depicts the differences and similarities between the samples linked to the sampling site.

### 3.6. Staff vs. Patient Toilets 

Almost one-tenth (9.16%, 24/262) of all microbiomes evaluated were from the staff sanitary inventory rather than from patients. These were exclusively samples collected from the TCH. They were 9.24% of those sequenced with the V1–V2 primer pair (16/172) and 8.99% of those sequenced with V3–V4 (8/89). In terms of species richness and diversity indices, large differences were evident between the two groups when limiting the analysis to the surgical wards. A significantly lower species richness (t(31.3) = −2.77, *p* = 0.009), and Shannon (t(46) = −2.53, *p* = 0.015) and Fisher-alpha diversity (t(32) = −2.52, *p* = 0.017) were observed across patient toilets. To confirm this observation without the bias introduced by quadruplication, we matched only samples stored in the same medium, extracted in the same way, and sequenced with the same primer pairs. The results are listed in Table 2. Although all analyses confirmed these results in their tendencies, only two were statistically significant. It should be considered that in all four analyses, the sample size did not exceed ten.

## 4. Discussion

Any microbiome analysis intends to reflect the microbial composition of the sample as faithfully as possible. The best, but not always the most practical, approach is to immediately process the sample or immediately freeze the sample until it is further processed [23,24]. In practice, preservation media are often used, chemically producing the effect otherwise achieved by freezing. Numerous studies have compared the performance of different preservation media against each other, against immediate freezing, and against native storage at room temperature [24]. However, while the vast majority have focused on stool samples, to our knowledge, no environmental swabs of indoor hospital environments have yet been studied in this regard. Given that the intent of our study was, among other things, to develop a protocol for collecting and processing hospital microbiome specimens, we only compared two preservation media with each other since it is not considered an option to routinely freeze samples upon collection or store them without preservation media. However, it would certainly be useful to compare preservation media with immediate freezing in a follow-up study to determine the extent of any potential differences. 

It is difficult to determine which of the extraction kits with their respective minor differences better reflects the actual conditions [25]. A welcome discovery was that the results differed only slightly. Further extraction kits and extraction modalities should be compared. To determine which one of the two primer pairs better reflects the actual composition of the sample, an additional metagenomic analysis should be performed. Currently, it would be difficult to choose between the V1–V2 primer, which seems to better detect *Pseudomonas*, and the V3–V4 primer, which seems to better detect *Escherichia*-*Shigella* and *Legionella*. If neither performs well when compared to metagenomics, other primer pairs would be needed for further investigation. Full-length 16S rRNA gene amplification analyses (16S-longreads) or metagenomic analyses would certainly deliver additional relevant information beyond the short-reads-based 16S amplificon analysis. Based on the cost–benefit ratio, it would have to be investigated in which cases they would bring more obvious advantages [6,26,27,28]. Additionally, it would be desirable to investigate which detected taxa are still viable [29], something for which culture-based methods are still vital in routine practice.

All in all, it seems reasonably viable to compare analyses of differently processed samples up to a certain extent. It seems more challenging to compare microbiomes from different hospitals and wards. Here, which taxa or variables are predictive of what must be investigated in more detail. Further investigations should also clarify which sampling locations are best for which applications. 

Most interesting is the species richness and diversity differences observed between patient and staff toilets on the surgical wards despite the small sample size. It should be confirmed forthwith whether patient toilets in wards where large amounts of antibiotics are prescribed are indeed characterized by lower species richness and diversity and that the lower diversity does not simply derive from an altogether worse health state of patients compared to staff. This would suggest that the emergence and maintenance of multidrug-resistant monocultures in hospital wastewater are linked to excreted antibiotics [30,31]. In a hospital setting, where patients are often immunosuppressed, it may be good to be exposed to an ecosystem with minimal microbial diversity. However, it is also possible that the lack of a rich, diverse microbiome may negatively impact patient outcomes. Without a diverse microbial community, pathogens that would otherwise be displaced could thrive [9,12,13,14,15,16,17,32,33].

## 5. Conclusions

The fight against antibiotic-resistant pathogens demands intensified monitoring of antimicrobial resistance. Next-generation sequencing has been used extensively for this purpose. Amplicon sequencing of genes such as the 16S rRNA gene offers excellent monetary value for the study of hospital microbiome profiles, leading to subsequent improvements in protocols. These molecular analyses could be integrated into ongoing surveillance programs. Our study provides evidence that protocol-related variability can be kept to a minimum and allows follow-up studies to address identified challenges.

## Figures and Tables

**Table 1:** Prevalence of taxa when sequenced with primer pair V1–V2 compared to V3–V4.

	Higher Prevalence	Lower Prevalence
Phylum	*Actinobacteriota* (t(126.06) = 6.44, *p* = < 0.001)	*Bacteroidota* (t(124.82) = −4.75, *p* = < 0.001)
		*Bdellovibrionota* (t(128.87) = −3.95, *p* = < 0.001)
		*Verrucomicrobiota* (t(103.03) = −5.55, *p* = < 0.001)
		*Acidobacteriota* (t(116.99) = −2.33, *p* = 0.021)
		*Chloroflexi* (t(106.62) = −3.78, *p* = < 0.001)
Class	*Actinobacteria* (t(119.62) = 6.73, *p* = < 0.001)	*Bacteroidia* (t(124.82) = −4.72, *p* = < 0.001)
		*Bdellovibrionia* (t(132.42) = −3.78, *p* = < 0.001)
		*Verrucomicrobiae* (t(104.34) = −4.5, *p* = < 0.001)
		*Plactomycetes* (t(137.82) = −2.28, *p* = 0.024)
Order	*Pseudomonadales* (t(141.41) = 2.21, *p* = 0.029)	*Enterobacterales* (t(108.68) = −4.71, *p* = < 0.001)
	*Corynebacteriales* (t(135.31) = 4.2, *p* = < 0.001)	*Flavobacteriales* (t(161.51) = −2.59, *p* = 0.011)
	*Propionibacteriales* (t(95.63) = 4.62, *p* = < 0.001)	*Cytophagales* (t(163.96) = −2.22)
	*Micrococcales* (t(156.14) = 2.23, *p* = 0.027)	*Chitinophagales* (t(118.58) = −3.31, *p* = 0.001)
	*Pseudonocardiales* (t(91.89) = 2.48, *p* = 0.015)	*Sphingobacteriales* (t(121.64) = −4.09, *p* = < 0.001)
		*Bdellovibrionales* (t(139.31) = −2.89, *p* = 0.005)
		*Acetobacterales* (t(120.77) = −3.45, *p* = 0.001)
		*Legionellales* (t(132.55) = −2.28, *p* = 0.024)
Family	*Pseudomonaceae* (t(129.75) = 2.38, *p* = 0.019)	*Enterobacteriaceae* (t(108.26) = −4.64, *p* = < 0.001)
	*Propionibacteriaceae* (t(94.67) = 4.74, *p* = < 0.001)	*Chitinophagaceae* (t(116.69) = −3.13, *p* = 0.002)
	*Hyphomicrobiaceae* (t(109.04) = 2.65, *p* = 0.009)	*Bdellovibrionaceae* (t(139.31) = −2.89, *p* = 0.005)
	*Microbacteriaceae* (t(114.06) = 2.93, *p* = 0.004)	*Flavobacteriaceae* (t(131.52) = −2.59, *p* = 0.011)
	*Mycobacteriaceae* (t(105.44) = 3.65, *p* = < 0.001)	*Sphingobacteriaceae* (t(120.42) = −3.41, *p* = 0.001)
	*Pseudonocardiaceae* (t(91.89) = 2.48, *p* = 0.015)	*Acetobacteraceae* (t(120.77) = −3.45, *p* = 0.001)
		*Legionellaceae* (t(132.55) = −2.28, *p* = 0.024)
Genus	*Pseudomonas* (t(130.47) = 2.31, *p* = 0.022)	*Escherichia-Shigella* (t(95.52) = −5.29, *p* = < 0.001)
	*Cutibacterium* (t(92.62) = 4.66, *p* = < 0.001)	*Sphingomonas* (t(179) = −2.25, *p* = 0.026)
	*Hyphomicrobium* (t(126.76) = 2.35, *p* = 0.02)	*Bdellovibrio* (t(139.19) = −2.87, *p* = 0.005)
	*Mycobacterium* (t(105.44) = 3.65, *p* = < 0.001)	*Flavobacterium* (t(131.16) = −2.71, *p* = 0.008)
	*Microbacterium* (t(105.42) = 3.5, *p* = 0.001)	*Legionella* (t(132.46) = −2.25, *p* = 0.026)
	*Pseudonocardia* (t(91.79) = 2.43, *p* = 0.017)	*Mesorhizobium* (t(88.44) = −3.09, *p* = 0.003)
	*Ochrobactrum* (t(95.55) = 2.55, *p* = 0.013)	
	*Acidovorax* (t(95.7) = 3.67, *p* = < 0.001)	
	*Shinella* (t(145.01) = 2.46, *p* = 0.015)	
	*Delftia* (t(152.05) = 2.18, *p* = 0.031)	
	*Amaricoccus* (t(112.84) = 2.28, *p* = 0.024)	
	*Ottowia* (t(99.21) = 2.43, *p* = 0.017)	
Species	*Lactobacillus iners* (t(92.48) = 2.61, *p* = 0.011)	
	*Microbacterium lacticum* (t(91) = 2.48, *p* = 0.015)	

**Table 2:** Prevalence of taxa when sequenced with primer pair V1–V2 compared to V3–V4; significant differences highlighted in green.

	V1–V2		V3–V4	
	ZBM n = 10	PMP n = 10	ZBM n = 10	PMP n = 10
Richness	t(3.17) = −0.95, *p* = 0.408	t(8) = −2.39, *p* = 0.044	t(3.02) = −1.5, *p* = 0.229	t(3.14) = −1.74, *p* = 0.177
Shannon diversity	t(3.11) = −0.97, *p* = 0.402	t(8)=−2.65, *p* = 0.029	t(8) = −1.63, *p* = 0.142	t(8) = −1.85, *p* = 0.101
Simpson diversity	t(8) = −0.37, *p* = 0.719	t(8) = −1.96, *p* = 0.086	t(8) = −1.12, *p* = 0.297	t(8) = −1.09, *p* = 0.308
Fisher-alpha diversity	t(3.1) = −1.04, *p* = 0.373	t(8) = −2.23, *p* = 0.056	t(3.02) = −1.46, *p* = 0.24	t(3.16) = −1.7, *p* = 0.183

## Data Availability

All data relevant to the study are included in the article.
